# Innovative Thermoplastics Composites Made from Recycled Poly(Propylene) Reinforced with Coconut Coir Fibers

**DOI:** 10.3390/polym18040432

**Published:** 2026-02-09

**Authors:** Arif Nuryawan, Nanang Masruchin, Raja Biandi Damanik, Iwan Risnasari, Hardiansyah Tambunan, Himsar Ambarita, Byung-Dae Park

**Affiliations:** 1 Faculty of Forestry, Universitas Sumatera Utara, 2nd Campus, Deli Serdang Regency 20135, North Sumatra, Indonesia; rajadamanik19@gmail.com (R.B.D.); iwanrisnasari@gmail.com (I.R.); 2 Research Center for Biomass and Bioproducts, National Research and Innovation Agency (BRIN), Jl. Raya Bogor Km. 46, Cibinong 16911, West Java, Indonesia; nana021@brin.go.id; 3 Faculty of Agriculture and Forestry, Universitas Satya Terra Bhinneka, Medan 20128, North Sumatra, Indonesia; htambunan911@gmail.com; 4 Faculty of Engineering, Universitas Sumatera Utara, Medan 20155, North Sumatra, Indonesia; himsar@usu.ac.id; 5 Department of Wood and Paper Sciences, Kyungpook National University, Daegu 41566, Republic of Korea; byungdae@knu.ac.kr

**Keywords:** thermoplastic composite, poly(propylene), coconut coir fibers, physical and mechanical properties

## Abstract

This study aims to evaluate the properties of poly(propylene) or PP composite reinforced with coconut coir fibers, and how these vary with fiber length and composition ratio. This innovative thermoplastic composite material was manufactured using a low-tech process from only PP, coconut coir fibers, and xylene (dissolution agent). Therefore, this process is widely accessible whilst both reusing/recycling waste plastic and making use of waste fiber material to produce a useful material that can fulfill demand for wood products, which has many environmental benefits. In this research, the coconut coir fibers are used as reinforcement, as well as the filler of the composite. Nine variations in composite material were produced from three length categories of fibers (2–5 mm, 10–20 mm, and 30–40 mm) and three composition ratios (60:40, 70:30, and 80:20) of predominant plastics of PP and fibers. Physical properties of the respective composite, such as density, moisture content, and thickness swelling, were fulfilled to the Japanese Industrial Standard (JIS) for particleboard. Mechanical properties of the composites showed that both modulus of elasticity (MoE) and modulus of rupture (MoR) decreased as the length of the fibers used increased. Conversely, an increase in the proportion of PP resulted in a stronger composite. However, statistically, the interaction between the amount of PP and the length of coir fibers within the biocomposite did not influence their quality. These results demonstrate that a low-cost process for manufacturing composite from waste materials can meet most industry standards and indicate that further refinement of the process, building on these findings, could achieve an innovative thermoplastic composite with widespread structural applications whilst delivering environmental benefits.

## 1. Introduction

The world produces twice as much plastic waste as it did two decades ago, with a majority ending up in landfills, being burned or dumped into the environment, and only 9% is being successfully recycled [1]. Plastic pollution, both macro and micro scales pose a problem as it has numerous detrimental effects on the environment and a high resistance to degradation, meaning it persists in the environment [2]. Efforts to discover sustainable methods for the disposal and reduction of plastic waste are still ongoing and are a key challenge facing the scientific community.

Poly(propylene) or PP is currently one of the most widely used types of thermoplastics, accounting for approximately 16.6% of global plastic production [3]. PP is frequently employed in applications as varied as packaging, construction, automotive, and sports equipment due to its favorable mechanical properties and affordable cost. Consequently, the generated PP waste can exhibit diverse characteristics and contaminants depending on its utilization [2]. At the end of its service life, PP waste can be processed through recycling or through disposal and incineration, which have harmful impacts on the environment and health, particularly due to the hazardous and toxic substances [4]. Recycling is predominantly achieved through mechanical processes, with the quality of recycled PP products depending on the physicochemical properties of PP, as well as processing conditions [5]. Ultimately, as recycling involves the collection and processing of waste plastic into new products, limiting the release of pollutants into the environment, it is a better alternative for end-of-life plastic products compared to disposal and incineration [6].

One possible utilization of PP waste is its incorporation into the production of other materials, such as innovative composite materials designed for non-structural applications, including outdoor decking, wall cladding, and lightweight building components, where moderate mechanical strength, dimensional stability, and moisture resistance are the key performance requirements. In recent years, composites with a thermoplastic matrix and bio-fillers or reinforcements from post-production organic waste materials have gained considerable interest. The characteristics of thermoplastic polymer materials allow for the addition of waste in various forms to the polymer matrix [7], where the resultant composite materials consist of the matrix phase and the reinforcement in the form of particles or fibers [8].

Studies over the past two decades have shown that composites serve as an alternative to many conventional materials due to a significant enhancement in the physical and mechanical properties of fiber-reinforced composite materials [9]. Natural fibers as reinforcements in polymer composites have garnered attention from numerous researchers due to their advantages, including low cost, abundance, non-abrasiveness, renewability, and biodegradability [10]. As composite materials can provide an alternative to wood, they can help meet the demand for wood products whilst also protecting forest environments [11]. Commonly used natural fibers derived from lignocellulosic materials include coir, jute, kenaf, hemp, sisal, kapok, and others [12].

Indonesia is home to the world’s largest coconut plantation area, covering 3.40 million hectares [13]. Currently, the industry primarily focuses on processing the coconut fruit for its flesh as the main product, while the handling of its byproducts, particularly coconut coir, is still done traditionally, low-cost processing, and requires optimization. Coconut coir consists of 75% fibers and 25% fine particles [14] and makes up a significant portion of the fruit, accounting for 35% of its total weight. With an average fruit production of 15.4 billion units per year in Indonesia and an estimated 1 million tons of the coir fiber produced annually [15,16], there is a substantial amount of biodegradable and renewable raw material available for making reinforcement of biocomposite products. Although thick and coarse, coir fibers exhibit durability and serve as good bolstering in the plastic matrix [17].

Several studies have been conducted on the development of composite materials using thermoplastic and coconut coir fiber. For instance, Naik’s research [18] investigated the mechanical properties of composites made from poly(ethylene) plastics and coconut fiber as a reinforcement, finding that the mechanical properties of the composite were competitive with plywood, making it an economical and viable alternative to plywood. Ayrilmis et al. [19] investigated the physical, mechanical, and flammability properties of coconut coir-reinforced PP composites with four ratios of fiber content, finding that the optimal composite panel formulation for the application of automotive interiors was obtained from a mixture of 60 wt% coir fiber, 37 wt% PP, and 3 wt% compatibilizer. In addition, Hussain et al. [20] investigated the mechanical properties of composite materials from a mixture of high-density poly(ethylene) or HDPE and coconut fiber, showing that the mechanical properties of the composite were strongly influenced by fiber length and composition ratio.

However, to date, there is no research focusing on the effect of the fiber length of coconut coir fiber on the properties of the composite with predominant PP. Therefore, this study aims to evaluate the physical and mechanical properties of thermoplastic composites made from predominant PP reinforced by coconut coir fiber and evaluate the effect of composition ratio and fiber length on the physical and mechanical properties of the resulting innovative composite.

This study addresses the valorization of lignocellulosic fiber waste in polypropylene composites, with a focus on understanding how fiber length, distribution, and concentration affect mechanical performance and dimensional stability. The selected processing parameters and characterization methods were chosen to provide a systematic evaluation of structure–property relationships, which are critical for the development of lightweight, low-density particleboard alternatives.

Further, unlike conventional particleboards made with thermosetting resins, our study explores the use of thermoplastic PP as a matrix for coir fiber composites. While thermoplastics do not cure like formaldehyde-based resins, PP-based composites can still provide sufficient mechanical strength and moisture resistance for certain particleboard-like applications, especially in lightweight, recyclable, or non-structural panels.

## 2. Materials and Methods

### 2.1. Materials

The materials used in this study were first recycled PP from beverage cup waste, which was cut into approximately 10 mm × 10 mm in size, coconut coir fiber of three length classes (2–5 mm, 10–20 mm, and 30–40 mm), NaOH 5% (*w*/*v*), and xylene. Both NaOH and xylene were technical and reagent grade, respectively and purchased from CV Rudang Jaya (chemical laboratory equipment, Medan, Indonesia). The use of xylene as a solvent was based on the investigation by Poulakis et al. [21], who demonstrated that xylene is a highly effective solvent system for the dissolution/precipitation technique in PP recycling. Xylene was used in this study as an effective and well-established reference solvent for the dissolution/precipitation processing of polypropylene, as demonstrated in prior work. While xylene is an organic solvent and its evaporation raises sustainability concerns, its use here is limited to investigating the feasibility and fundamental behavior of this processing approach rather than proposing an industrially sustainable route. Conventional thermal processing methods, such as extrusion, remain more suitable for large-scale applications, with the solvent-based approach serving as a complementary method primarily for research purposes or cases where thermal processing is constrained.

### 2.2. Preparation of Thermoplastic Composites Raw Materials

The coconut coir fiber was dried under sunlight and further processed with alkali treatment, also referred to as delignification [22]. This process enhances the quality of PP composites by exposing more cellulose on the fiber surface, which increases surface roughness, improves fiber–matrix adhesion, and promotes better stress transfer and uniform fiber dispersion, in the hope of achieving improved mechanical performance of the composite. The fiber was first immersed in a NaOH 5% (*w*/*v*) solution at 80 °C and stirred for 1 h. The fiber was then added as much as 70 g per 1 L of alkaline solution, before it was filtered and washed with clean water until the alkalinity was removed. Afterward, it was dried at 100 °C until a constant weight was achieved. Once processed, the coconut fiber was stored in an airtight container to maintain its moisture content (MC).

The thermoplastic of PP was washed with water until clean, then dried in the sunlight. The dried PP was then dissolved in xylene in a glass reactor equipped with a stirrer and mantle heater, with a ratio of 1:6.7 (*w*/*v*) at a temperature of 135 °C [21]. The resulting solution was mixed with the coconut fibers according to the predetermined length classes and the plastic-to-fiber ratios (based on weight), namely 60:40, 70:30, and 80:20. The mixture was then stirred for 1 h to ensure homogeneity. Subsequently, it was transformed into pellets and placed in an acid chamber overnight to evaporate the xylene [23].

### 2.3. Fabrication of Thermoplastic Composites

The target density of the composite is 0.70 g/cm^3^ with dimensions of 25 cm × 25 cm × 1 cm. The process for molding each specimen was carried out using a hot press at a temperature of 160 °C for 10 min and a pressure of 2.94 MPa. In the hot-pressing process, the mold was first placed in the hot press and heated to 160 °C under a low contact pressure of approximately 0.49 MPa to allow gradual heating and air release from the composite mat. After reaching the target temperature, the pressure was then increased to 2.94 MPa and maintained for 10 min to ensure proper consolidation. This stepwise pressure application was intended to minimize trapped air and reduce the risk of gas bubble formation and microcrack development. No additional vacuum-assisted deaeration was applied during pressing.

Immediately after being pressed, the composite was still hot and soft, and was therefore allowed to cool and harden for at least 120 min, prior to removing it from the mold. To equalize the MC of the composite and release internal residual stress resulting from the heat compression, conditioning was conducted for one week. The mass of raw materials used in the manufacture of each thermoplastic composite for different composition ratios and coir fiber length is shown in Table 1.

### 2.4. Testing of Thermoplastic Composite Properties

The thermoplastic composites were cut and tested following the Japanese Industrial Standard for particleboards (JIS A 5908-2003) [24]. Since the composites are closer in form to waferboard (type 17.5–10.5) than to Oriented Strand Board/OSB (type 24–10), the standards for waferboard were applied. Tests included physical properties—density, MC, water absorption, and thickness swelling—as well as mechanical properties, specifically modulus of elasticity (MoE) and modulus of rupture (MoR). Density and MC testings were conducted using samples with dimensions of (10 × 10 × 1) cm^3^. Density was calculated from measured mass and volume of the sample, while the MC was determined by gravimetric analysis by measuring the initial and final mass of the sample after drying it in an oven at a temperature of 103 ± 2 °C for 24 h. Testing for water absorption and thickness swelling using samples with dimensions of (5 × 5 × 1) cm^3^ was carried out by immersing the sample in water for 24 h. Water absorption was determined by measuring the difference in mass before and after immersion, while the thickness swelling was determined by measuring the change in thickness of the sample before and after immersion. Meanwhile, mechanical testing (MoE and MoR) was conducted using samples with dimensions of (20 × 5 × 1) cm^3^ using the Tensilon Universal Testing Machine (UTM) (UTM Tensilon RTF-1350, A&D Company, Tokyo, Japan) with a crosshead speed of 10 mm/min.

### 2.5. Fourier Transform Infrared (FTIR) Spectroscopy

The infrared spectrum of the thermoplastic of PP and the resulting thermoplastic composite materials were recorded using the FTIR-UATR Perkin Elmer Spectrum Two (Waltham, MA, USA). The FTIR wavenumber was in the range of 4000–400 cm^−1^ with 16 scans for each case at a resolution of 4 cm^−1^.

### 2.6. Morphology of the Biocomposite

The morphology of the thermoplastic composite was observed using a photomicroscope (Keyence VHX Digital Microscope VHX-6000, Keyence Corporation, Osaka, Japan). Both widthwise and lengthwise views were captured at different magnifications to evaluate the performance of the resulting thermoplastic composites.

### 2.7. Analysis of Statistics

Statistical analysis was performed using a factorial Completely Randomized Design (CRD) with 2 factors, namely fiber length (2–5 mm, 10–20 mm, and 30–40 mm) and PP/fiber ratio (60:40, 70:30, and 80:20). If the treatment had a significant effect at *p*-value < 0.05, then the Duncan Multi Range Test (DMRT) was performed to determine the level of treatment that was significantly different. A total of 3 replications for each treatment were used in this study; therefore, there were 27 thermoplastic composite boards in total.

## 3. Results and Discussions

### 3.1. Density

**Figure 1:**
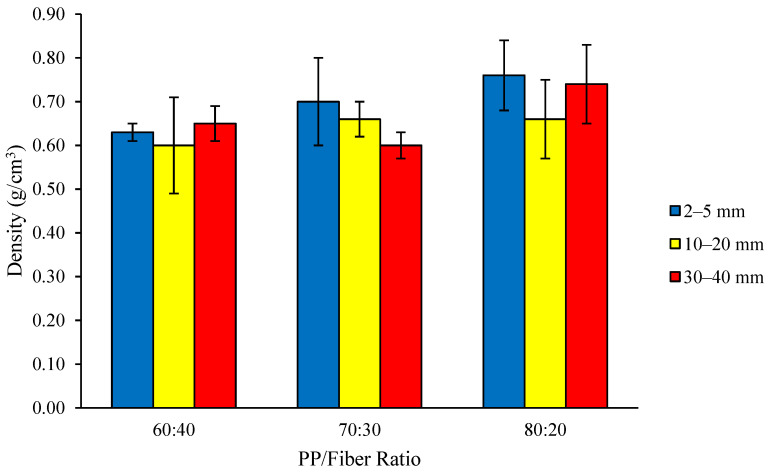
Density of the thermoplastic composites made of PP reinforced by coconut coir fiber.

The density of the thermoplastic composite ranges from 0.60 to 0.76 g/cm^3^ (Figure 1). The highest density value (0.76 g/cm^3^) was found with fiber lengths of 2–5 mm and a PP/fiber ratio of 80:20, while the lowest density (0.60 g/cm^3^) was found for fiber lengths of 10–20 mm and 30–40 mm and a PP/fiber ratio of 60:40 and 70:30. The density of all composites met the JIS A 5908–2003 standard (density of 0.40–0.90 g/cm^3^); however, most did not meet the target of fabrication of the board which was 0.70 g/cm^3^. Our values were comparable with those achieved for the composite from PP and cotton stalk of 0.70 g/cm^3^ [25]. Although the PP and fiber masses were precisely measured and the mold geometry was constant, small differences in sample thickness, compaction, or trapped air/voids during processing, as well as slight variations in fiber distribution, can naturally lead to the observed range of densities. The achieved density is closely related to the distribution of coconut coir fiber sizes in the composite system; smaller fibers can establish closer contact and stronger bonding with the plastic at the interface, resulting in higher-density composites [26].

**Figure 2:**
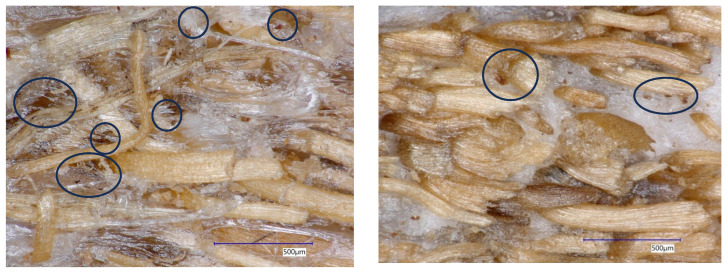
There are more pores and voids as depicted in the picture as black circles within the thermoplastic composites made of longer fiber (**left**) compared to the one made of shorter fiber (**right**).

In this study, the achieved density is influenced by the number and size of pores, or void spaces, within the composite material. Analysis of variance showed that only the composition ratio had a significant effect on density, while the DMRT revealed that the composite with an 80:20 PP/fiber ratio differed from the 60:40 ratio but was not statistically different from the 70:30 ratio. Neither fiber length nor its interaction with composition had a statistically significant impact on density. These results highlight that density is primarily governed by the PP/fiber ratio rather than fiber length. The significantly higher density observed for the 80:20 compared to the 60:40 PP/fiber ratio suggests that the higher fiber content in the 60:40 composite leads to closer packing of fibers, which can prevent complete infiltration of PP into the matrix and result in more void spaces. Although not statistically significant, microscope observations suggest that composites made from longer fibers with higher fiber content tend to have more and larger pores, whereas those made with shorter fibers exhibit higher compaction and fewer voids [27] (Figure 2). Furthermore, the achieved density is closely related to the distribution of coconut coir fiber sizes in the composite; smaller fibers can establish closer contact and stronger bonding with the plastic at the interface, leading to higher-density composites [26]. These findings reflect a common challenge in thermoplastic composite development, namely the interfacial interaction between fibers and plastic, where imperfect bonding can result in gaps at the interface [11].

### 3.2. Moisture Content (MC)

**Figure 3:**
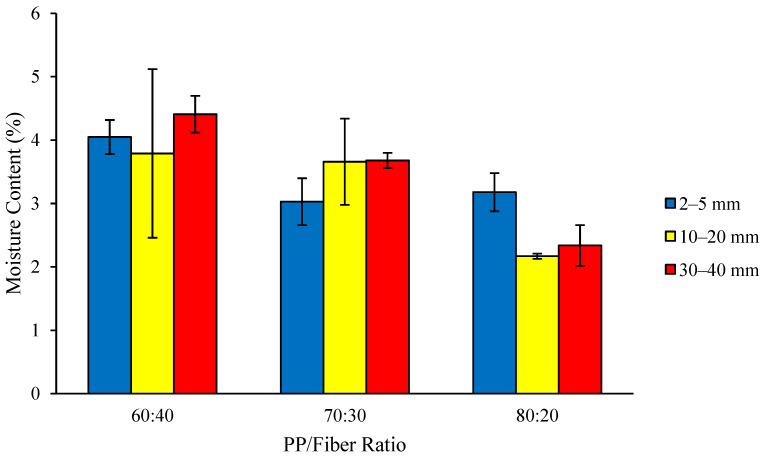
MC of the thermoplastic composites made of PP reinforced by coconut coir fiber.

Thermoplastic composite’s MC ranges from 2.17 to 4.41% (Figure 3), with the highest MC (4.41%) found in the composite with a fiber length of 30–40 mm and a PP/fiber ratio of 60:40, while the lowest value (2.17%) was found in the composite with a fiber length of 10–20 mm and a PP/fiber ratio of 80:20. The measured MC of 2.17–4.41% is mainly due to the hydrophilic nature of coconut coir fibers rather than the polypropylene (PP) matrix. Despite prior drying and hot pressing at 160 °C, the fibers can retain bound moisture within their cell walls and can reabsorb moisture from ambient air during handling before processing. Hot pressing removes free surface moisture but does not fully eliminate chemically bound or strongly absorbed water, which remains in the composite. The MC values of the composite met the JIS A 5908-2003 standard, which specifies a maximum MC of 14%. The low MC is attributed to the hydrophobic nature of the plastic, which is the main component of the composites [24].

An increase in hydrophobic PP combined with a decrease in hydrophilic coconut fiber led to a reduction in moisture content. The analysis of variance indicates that there are no significant effects of fiber length or composition ratio on moisture content (MC), either individually or through their interaction. The overall low moisture content of the thermoplastic composite is due to two factors. Firstly, the nature of the composite material results in relatively few pores, with the PP filling most spaces between fibers during manufacture, leaving little available pore space for ambient moisture to infiltrate [28]. Additionally, the delignification process performed here on the fiber raw material, resulting in the removal of lignin present in the fiber raw material, leads to decreased water absorption in fibers.

### 3.3. Water Absorption

**Figure 4:**
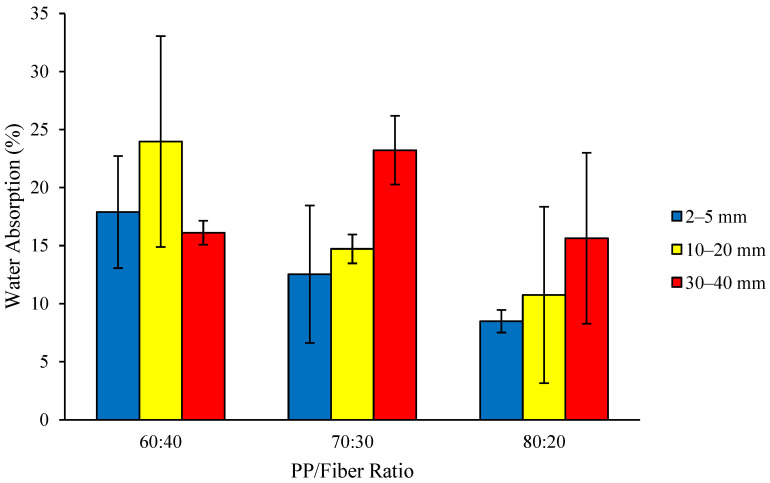
Water absorption of the thermoplastic composites made of PP reinforced by coconut coir fiber.

Water absorption of the thermoplastic composite ranges from 8.49 to 23.97% (Figure 4). The highest water absorption (23.97%) was found in the composite with a fiber length of 10–20 mm and a PP/fiber ratio of 60:40, whilst the lowest water absorption (8.49%) was found in the composite with a fiber length of 2–5 mm and a PP/fiber ratio of 80:20. Although the water absorption values presented in Figure 4 are relatively high, a consistent trend is evident: increasing both fiber content and fiber length leads to higher water absorption. This behavior can be attributed to the absence of coupling agents in this study, which results in insufficient interfacial wetting between the hydrophobic PP matrix and the hydrophilic coconut coir fibers, thereby increasing water uptake.

Water absorption is an important physical property of composite materials; however, JIS A 5908-2003 does not provide an ideal criterion for water absorption value to compare because this standard was used only for consistency in testing methodology rather than for compliance with a specific acceptance limit.

Consistent with the trend observed for MC, water absorption decreased as the proportion of hydrophobic PP increased and hydrophilic coconut fiber decreased. The analysis of variance results indicated that the interaction between fiber length and raw material ratio, as well as a single factor, had no significant effect on the water absorption of the thermoplastic composite. In general, higher-density composites (Figure 1) exhibit lower water absorption than lower-density composites which is expected since low-density materials contain a greater proportion of void spaces that allow water infiltration. However, several additional factors will influence the water absorption of a given composite, including the volume of voids that can accommodate water, the outer surface of the plastic composite that cannot be covered by the polymer matrix, and the depth of polymer molecule penetration into the fibers [29]. Additionally, results show water absorption increased with the increasing proportion of coconut coir fiber; this is due to the hydrophilic nature of the coir fiber, and the increased water absorption originates from composites with a higher fiber content [30].

### 3.4. Thickness Swelling

**Figure 5:**
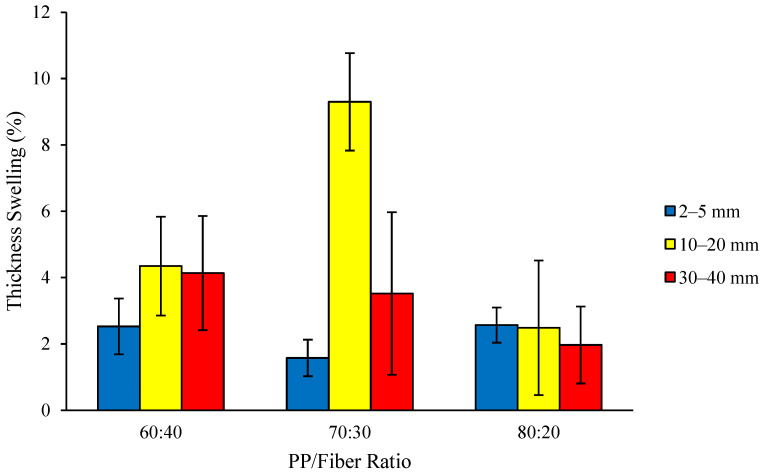
Thickness swelling of the thermoplastic composites made of PP reinforced by coconut coir fiber.

As shown in Figure 5, the thickness swelling of the thermoplastic composites ranged from 1.58 to 9.30%, with all values meeting the JIS A 5908-2003 requirement (≤12%). At a PP/fiber ratio of 70:30, the lowest and highest thickness swelling values were observed for composites reinforced with 2–5 mm fibers (1.58%) and 10–20 mm fibers (9.30%), respectively. This information supports the idea that the thickness swelling of the PP/fiber composites was not governed solely by the fiber length, but also by other factors such as fiber content, fiber dispersion quality, and interfacial bonding.

Further, the analysis of variance results indicated that the interaction between fiber length and raw material ratio, as well as a single factor, had no significant effect on the thickness swelling of the composites. According to Sotannde et al. [31], water absorption and thickness swelling are physical properties related to the dimensional stability of composites. Generally, higher water absorption leads to increased thickness swelling, and vice versa. Thickness swelling is related to water absorption, where the more water is absorbed and enters the composite structure, the greater the dimensional changes generated. Although longer fibers are generally expected to increase thickness swelling by creating more voids within the composite structure [32], the present findings contradict this assumption. The highest thickness swelling occurs with medium fiber lengths (10–20 mm), whereas composites with the longest fibers (30–40 mm) show comparatively lower swelling. This deviation suggests that additional factors, such as fiber dispersion, interfacial adhesion, and the degree of composite compaction, significantly affect swelling behavior. Therefore, fiber length alone does not determine thickness swelling in these thermoplastic composites.

### 3.5. Modulus of Elasticity (MoE)

**Figure 6:**
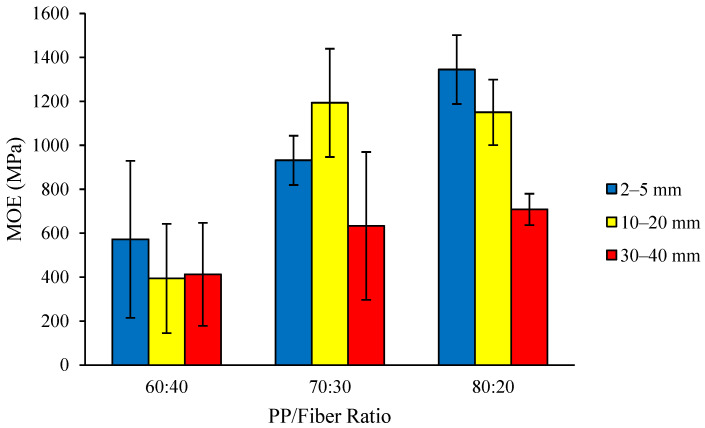
MoE of the thermoplastic composites made of PP reinforced by coconut coir fiber.

The MoE of thermoplastic composites ranges from 394 to 1345 MPa (Figure 6). The highest MoE value (1345 MPa) was found in the composite with a fiber length of 2–5 mm and a plastic/fiber ratio of 80:20, while the lowest MoE value (394 MPa) was found in the one with a fiber length of 10–20 mm and a plastic/fiber ratio of 60:40. None of the MoE values obtained meet the requirement of JIS A 5908-2003, which states a value of ≥2001 MPa. However, despite not fulfilling the JIS A 5908-2003 requirement, our results for the 80:20 PP/fiber ratio composites are comparable with both the MoE of PP (1530 MPa) and the lower end of the range of MoE for USA industrial particleboard products (1600–3700 MPa) [33]. The relatively low MoE values may be attributed, at least in part, to residual porosity arising from the absence of a vacuum deaeration step during processing, which can limit effective stress transfer between the matrix and fibers. Conventional particleboard differs from thermoplastic composites in material composition and processing, particularly in the type of binding agent (adhesive versus polymer matrix) and the polymer-to-lignocellulosic material ratio, which is limited in particleboard but higher in thermoplastic composites.

**Figure 7:**
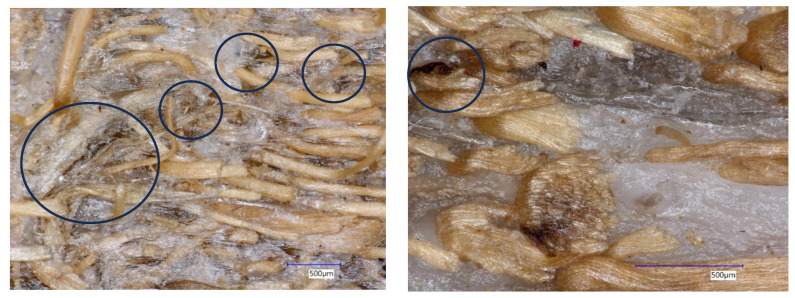
Cracks as shown in black circles within the thermoplastic composites leading to values of MoE were lower compared to the standard; low magnification on the composite with long fibers explained this condition (**left**), and high magnification on the composite showed greater cracks in the interphase between the PP matrix and the reinforcement fiber (**right**).

The low values of MoE obtained were attributed to several factors, one of which is the incomplete bonding of the composite surface, leading to cracks on its sides as depicted in Figure 7.

Lu [34] reported that crack formation in thermoplastic composites is strongly influenced by the local fiber packing density and the degree of fiber–matrix consolidation along the fiber orientation. Incomplete bonding of the constituent materials during the compression process can lead to the formation of microscopic cracks, which may not be visible on the smooth composite surface but can be observed at the edges under high magnification (Figure 7). During MoE testing, the presence of such cracks reduces the effective load transfer within the composite, resulting in decreased bending stiffness. In addition, the use of fibers with a wide length distribution can lead to a non-uniform fiber distribution across the composite sheet, causing spatial variations in interfacial bonding and further reducing bending strength [35].

The analysis of variance results indicated the interaction between fiber length and raw material ratio, and the single factor of fiber length had no significant effect on the MoE of the composite, while the single factor of raw material ratio had a significant effect. The composites with a higher proportion of PP exhibit higher MoE values. Youssef et al. [36] stated that increasing the percentage of plastic in the mixture enhances the interfacial adhesion between the matrix and the fibers, resulting in a more uniform distribution of applied stress and consequently increasing the absorbed energy until failure. This is reflected in our results, with previous sections discussing the greater proportion of void spaces and lower density of composites with a lower proportion of PP and DMRT results for MoE (Table 2) showing the PP/fiber ratio of 80:20 was significantly different from the PP/fiber ratios of 60:40 and 70:30.

### 3.6. Modulus of Rupture (MOR)

**Figure 8:**
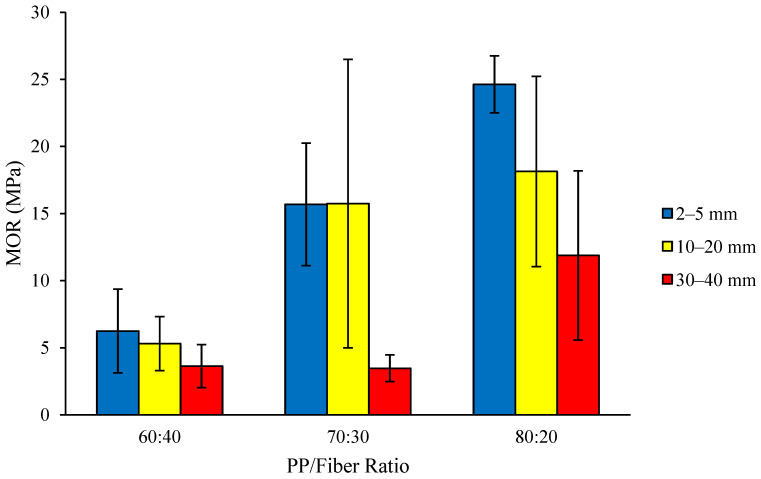
MOR of thermoplastic composites made of PP reinforced by coconut coir fiber.

The value of MoR of thermoplastic composite ranges from 3.48 to 24.63 MPa (Figure 8), with the highest MoR value (24.63 MPa) found for a fiber length of 2–5 mm and a PP/fiber ratio of 80:20, while the lowest MoR value (3.48 MPa) was found for a fiber length of 30–40 mm and a PP/fiber ratio of 70:30. Most of the composites of PP/fiber ratios of 80:20 and 70:30 produced MoR values which met the JIS A 5908-2003 standard (≥8.04 MPa), although all composites of PP/fiber ratio of 60:40 did not meet the standard.

The analysis of variance results indicated that the interaction between fiber length and raw material ratio had no significant effect on the MoR of the thermoplastic composite, but the two single factors had a significant effect. Similarly, to the MoE values, the MoR values obtained indicated that the higher the ratio of plastic compared to fiber raw material, the higher the resulting MoR value. The MoR value is also directly proportional to density, meaning that the higher the density of the board, the higher the MoR value of the composite [37]. It is also known that an increase in fiber length leads to a low value of MoR, with the findings reported by Gulitah and Liew [38] stating that increasing fiber size causes poor interfacial bonding between the fiber and plastic matrix. When the fiber cannot bond well with the plastic matrix, it results in voids that reduce the strength of the composite. DMRT results (Table 2) showed that the thermoplastic composites with a fiber length of 2–5 mm were significantly different from the composite with a fiber length of 30–40 mm, but not significantly different from the composite with a fiber length of 10–20 mm. Additionally, the PP/fiber ratio of 80:20 was significantly different from the PP/fiber ratios of 60:40 and 70:30, mirroring the results obtained for MoE.

The values obtained for the 80:20 PP/fiber ratio and fiber lengths of 2–5 mm and 10–20 mm are comparable with the range of values found for US industrial particleboard products of 15.20–22.85 MPa [33], with the highest MoR value exceeding these. This shows that optimized PP/fiber thermoplastic composites using coconut coir fibers are able to retain much of the high flexural strength of PP.

All fiber lengths used in this study—short, medium, and long—exceed the critical fiber length required for effective stress transfer from the polypropylene matrix to the coir fibers. Consequently, variations in fiber length had little effect on the mechanical properties, as even the shortest fibers were sufficient to fully reinforce the composites.

### 3.7. FTIR Spectroscopy

FTIR analysis was conducted to examine the chemical stability of PP during composite processing and to confirm the presence of characteristic functional groups associated with alkali-treated fibers after composite fabrication. The FTIR spectra of the polymer (PP) and thermoplastic composites in each treatment are presented in Supplementary Figure S1. All thermoplastic composite spectra are similar, each prominently displaying characteristic peaks of PP, which is expected given that PP makes up the majority of material by mass in the composites, indicating that the addition of coconut fibers of different sizes does not substantially affect the chemical structure of the PP component of the composites.

As no compatibilizers or chemical coupling agents were used, no new absorption bands indicative of chemical reactions between PP and the fibers were expected. The FTIR spectra of the composites exhibited the characteristic peaks of PP along with fiber-related functional groups, indicating that melt processing and hot pressing did not induce chemical degradation or interfacial covalent bonding. These results suggest that the mechanical behavior of the composites is governed primarily by physical interfacial interactions, fiber dispersion, and consolidation quality, rather than by chemical modification of the matrix–fiber interface.

For all thermoplastic composites spectra in Supplementary Figure S1a–c, prominent peaks were observed at 2950, 2918, and 2838 cm^−1^ (CH_3_ and CH_2_ stretching) and at 1455 and 1376 cm^−1^ (methyl group umbrella modes) [39,40]. All composites also exhibited peaks at 1167 cm^−1^ and 900–800 cm^−1^, corresponding to C–O–C bonds in the pyranose ring and CH deformation of glycosidic bonds in cellulose [41,42]. Composites with longer fibers and higher fiber contents (PP/fiber ratios of 70:30 and 60:40) showed additional broad peaks between 1200 and 600 cm^−1^, indicative of various C–H, C–O, and O–H vibrations in cellulose [43], and broad peaks from 3500 to 3200 cm^−1^ due to absorbed water weakly bound to cellulose [40]. Small peaks at 1620–1642 cm^−1^, absent in other spectra, further confirmed the presence of water, while the absence of peaks at 1736–1719 cm^−1^ indicated successful delignification and negligible lignin/hemicellulose contributions [44,45].

Overall, PP characteristic peaks remained dominant, while fiber-related peaks (O–H, C=O, and C–O–C) increased with higher fiber content (60:40) and decreased at higher polymer ratios (80:20). In this thermoplastic composite, short fibers exhibited stronger polymer–fiber interactions (slight peak shifts and broadening), medium fibers showed moderate contributions, and long fibers displayed weaker fiber peaks due to agglomeration. These observations confirm that both fiber content and length influence FTIR band intensities and shifts, reflecting the degree of dispersion and interfacial bonding in the thermoplastic composites.

## 4. Conclusions

The results of this study demonstrate that the physical properties of thermoplastic composites made of PP and coconut coir fiber, such as density, moisture content, water absorption, and thickness swelling, have met the JIS A 5908-2003 standard. Additionally, most composites have MoR values that meet the required standard, although MoE values do not. The interaction between fiber length and the raw material ratio did not significantly affect the physical and mechanical properties of the resulting thermoplastic composites, although results indicate shorter fiber lengths and higher proportions of PP in the PP/fiber ratio may lead to improvements in properties. FTIR results confirm there is a degree of variability in manufacturing the composites, which indicates there is scope for further optimizing the production process and further improving thermoplastic composite properties. Although water content and water absorption were low compared to industrial standards, where present, this was attributed to microscopic cracks caused by imperfect bonding at the fiber/PP interface. The challenge of improving thermoplastic and fiber bonding strength in thermoplastic composites is an active area of research, and developments in methods will further enhance the quality of coconut coir fiber composite manufacture and properties.

Our combined results showed that a low-cost method of producing thermoplastic composites from waste materials can satisfy the majority of industry standards for particleboard, whilst delivering environmental benefits in terms of recycling, protecting forests by fulfilling demand for wood materials from other sources, and reducing the emission of harmful chemicals associated with conventional particleboard manufacture. Additionally, by building on these discoveries, further process optimization may be able to produce a composite with a wide range of structural applications and environmental advantages.

## Figures and Tables

**Table 1:** The amount of plastic and fiber raw materials.

Fiber Length	Ratio	PP (g)	Fiber (g)
2–5 mm	60:40	275.62	183.75
	70:30	321.56	137.81
	80:20	367.50	91.87
10–20 mm	60:40	275.62	183.75
	70:30	321.56	137.81
	80:20	367.50	91.87
30–40 mm	60:40	275.62	183.75
	70:30	321.56	137.81
	80:20	367.50	91.87

**Table 2:** DMRT results for each of the thermoplastic composite properties.

	Density	MoE	MoR
Fiber Length			
2–5 mm			6.34 a
10–20 mm			11.42 ab
30–40 mm			15.52 b
PP Fiber/Ratio			
60:40	0.63 a	459.70 a	5.07 a
70:30	0.65 ab	758.08 a	9.99 a
80:20	0.72 b	1183.42 b	18.22 b

The same letters in the values for each thermoplastic composite property are not significantly different based on the DMRT at a 5% significance level.

## Data Availability

The original contributions presented in this study are included in the article/Supplementary Material. Further inquiries can be directed to the corresponding author.

## Supplementary Materials

The following supporting information can be downloaded at: https://www.mdpi.com/article/10.3390/polym18040432/s1, Figure S1. FTIR spectrum of polymer (PP) and thermoplastic composites: (a) short, (b) medium, and (c) long fibers (1: PP/fiber ratio of 60:40, 2: PP/fiber ratio of 70:30, 3: PP/fiber ratio of 80:20).
