# Supplementary material for: Innovative Thermoplastics Composites Made from Recycled Poly(Propylene) Reinforced with Coconut Coir Fibers
Source: Polymers (Basel). 2026 Feb 9;18(4):432. doi: 10.3390/polym18040432 (PMC12943887; doi:10.3390/polym18040432)
Supplement: Supplementary file 1 [file polymers-18-00432-s001.zip › polymers-4103682-supplementary.pdf]

Supplementary materials

(a)

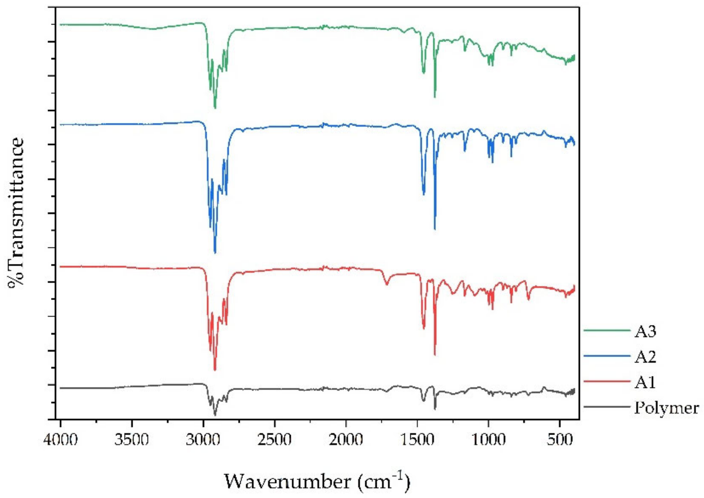

(b)

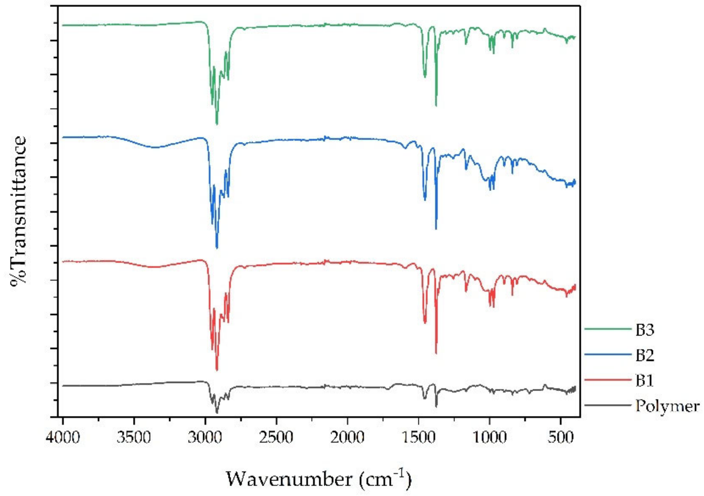

(c)

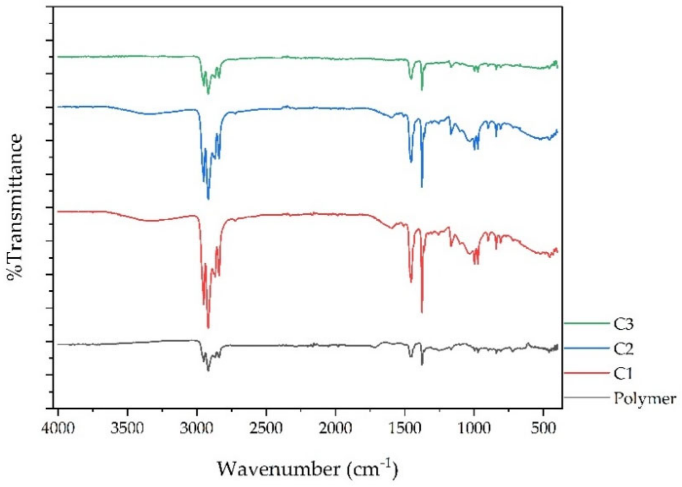

Figure S1. FTIR spectrum of polymer (PP) and thermoplastic composites: (a) short, (b) medium, and (c) long fibers (1: PP/fiber ratio of 60:40, 2: PP/fiber ratio of 70:30, 3: PP/fiber ratio of 80:20).

Supplementary materials

For all thermoplastic composites spectra in Figure S1 (a, b, c), prominent peaks were observed at 2950 cm^{-1}, 2918 cm^{-1}, and 2838 cm^{-1}, as well as at 1455 cm^{-1} and 1376 cm^{-1}, respectively indicating CH_3 stretching, CH_2 stretching, and methyl group umbrella mode, which were characteristic of PP [1,2]. All composite spectra also showed peaks at 1167 cm^{-1} and peaks ranging from 900–800 cm^{-1}, each indicating the C-O-C bond of pyranose ring skeletal and CH deformation of glycosidic bonds, respectively, characteristics of the β-glycosidic linkage between anhydro glucose units in cellulose [3,4].

Additionally, some spectra, notably those for longer fiber lengths and PP/fiber ratios of 70:30 and 60:40 (Figure S1, a) A3, b) B1, B2, and c) C1, C2) show additional, broad peaks from 1200 – 600 cm^{-1} which are characteristic of a range of C-H, C-O and O-H stretching and deformation in cellulose [5]. There are a range of broad peaks from 3500 – 3200 cm^{-1} in a) A3, b) B1, B2, and c) C1 and C2, not present in PP, and absent or only of very weak intensity in other spectra, these are due to absorbed water weakly bound to cellulose [2]. This is further confirmed as these same spectra show small peaks at 1620 – 1642 cm^{-1}, absent in other spectra which correspond to H-O-H bending in water [6]. FTIR confirms the delignification process was successful as there is an absence of characteristic peaks in at 1736 – 1719 cm^{-1} which correspond to C=O stretching in lignin and hemicellulose [7], and further confirms the peaks at 3500 – 3200 cm^{-1} are due to absorbed water rather than the stretching vibration of hydroxyl (OH) groups from lignocellulosic materials which also occur in this region [3,7].

In short, the FTIR spectra of PP and its fiber-reinforced composites show that characteristic PP peaks (C–H stretching, CH_2/CH_3 bending) remain dominant, while fiber-related peaks (O–H, C=O, C–O–C) increase with higher fiber content (60:40) and decrease at higher polymer ratios (80:20). Short fibers exhibit stronger polymer-fiber interactions, seen as slight peak shifts or broadening, medium fibers show moderate fiber contributions, and long fibers display weaker fiber peaks due to agglomeration. Overall, fiber content and length influence the intensity and subtle shifts of FTIR bands, reflecting the degree of dispersion and interfacial bonding.

Supplementary materials

References:

1. Fang, J.; Zhang, L.; Sutton, D.; Wang, X.; Lin, T. Needleless melt-electrospinning of polypropylene nanofibres. *J. Nanomater*. **2012**, *2012*, 1-9. https://doi.org/10.1155/2012/382639
2. Smith, B.C. The Infrared Spectra of Polymers III: Hydrocarbon Polymers. *Spectrosc*. **2021**, *36*, 22–25. https://doi.org/10.56530/spectroscopy.mh7872q7
3. Moradbak, A.; Tahir, P.M.; Mohamed, A.Z.; Abdi, M.M.; Razalli, R.L.; Halis, R. Isolation of cellulose nanocrystals from *Gigantochloa scortechinii* ASAM pulp. *Eur. J. Wood Wood Prod*. **2017**, *76*, 1021–1027. https://doi.org/10.1007/s00107-017-1244-1
4. Verma, D.; Gope, P.C. The use of coir/coconut fibers as reinforcements in composites. In *Biofiber Reinforcements in Composite Materials*, 1st ed.; Faruk, O., Sain, M., Eds.; Woodhead Publishing: Sawston, Cambridge, UK, 2015; pp. 285–319. https://doi.org/10.1533/9781782421276.3.285
5. Popescu, M.C., Popescu, C.M., Lisa, G. and Sakata, Y., 2011. Evaluation of morphological and chemical aspects of different wood species by spectroscopy and thermal methods. *Journal of molecular structure, 988*(1-3), pp.65-72.
6. Poletto, M., Pistor, V., Zeni, M. and Zattera, A.J. (2011) Crystalline Properties and Decomposition Kinetics of Cellulose Fibers in Wood Pulp Obtained by Two Pulping Processes. Polymer Degradation and Stability, 96, 679-685.
7. Hospodarova, V.; Singovszka, E.; Stevulova, N. Characterization of cellulosic fibers by FTIR spectroscopy for their further implementation to building materials. *Am. J. Anal. Chem*. **2018**, *9*, 303–310. https://doi.org/10.4236/ajac.2018.96023
